# Diethyl 1,1-dioxo-3,5-bis­(pyridin-2-yl)-1λ^6^,4-thio­morpholine-2,6-dicarbox­ylate

**DOI:** 10.1107/S1600536811000444

**Published:** 2011-01-08

**Authors:** P. Sugumar, N. Edayadulla, P. Ramesh, P. Ramesh, M. N. Ponnuswamy

**Affiliations:** a Centre of Advanced Study in Crystallography and Biophysics, University of Madras, Guindy Campus, Chennai 600 025, India; b Department of Natural Products Chemistry, School of Chemistry, Madurai Kamaraj University, Madurai 625 021, India

## Abstract

The title compound, C_20_H_23_N_3_O_6_S, crystallizes with two crystallographically independent mol­ecules in the asymmetric unit. The thio­morpholine ring in both mol­ecules adopts a chair conformation. The crystal structure is stabilized by C—H⋯O inter­actions. The amino groups are shielded and, as a result, these groups are not involved in hydrogen bonding.

## Related literature

For general background to quinoline derivatives, see: Katritzky *et al.* (1985 ▶); Ramana Reddy *et al.* (1990 ▶); Bhaskar *et al.* (2000 ▶). For the synthesis, see: Baliah & Rangarajan *et al.* (1954 ▶). For hydrogen-bond motifs, see: Bernstein *et al.* (1995 ▶). For puckering parameters, see: Cremer & Pople (1975 ▶). For asymmetry parameters, see: Nardelli (1983 ▶)
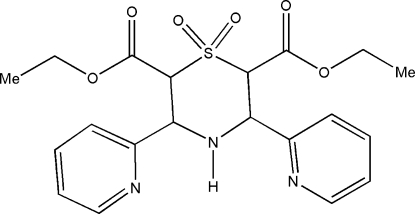


## Experimental

### 

#### Crystal data

C_20_H_23_N_3_O_6_S
*M* *_r_* = 433.47
Orthorhombic, 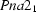

*a* = 20.7258 (13) Å
*b* = 8.3921 (5) Å
*c* = 24.4923 (15) Å
*V* = 4260.0 (5) Å^3^
*Z* = 8
Mo *K*α radiation
μ = 0.19 mm^−1^
*T* = 293 K
0.20 × 0.18 × 0.16 mm

#### Data collection

Bruker SMART APEXII area-detector diffractometer
Absorption correction: multi-scan (*SADABS*; Bruker, 2008 ▶) *T*_min_ = 0.962, *T*_max_ = 0.970
22898 measured reflections
9897 independent reflections
7453 reflections with *I* > 2σ(*I*)
*R*_int_ = 0.027

#### Refinement

*R*[*F* ^2^ > 2σ(*F* ^2^)] = 0.042
*wR*(*F* ^2^) = 0.113
*S* = 1.03
9897 reflections
553 parameters
1 restraint
H atoms treated by a mixture of independent and constrained refinement
Δρ_max_ = 0.29 e Å^−3^
Δρ_min_ = −0.17 e Å^−3^
Absolute structure: Flack (1983 ▶), 4584 Friedel pairs
Flack parameter: 0.14 (6)

### 

Data collection: *APEX2* (Bruker, 2008 ▶); cell refinement: *SAINT* (Bruker, 2008 ▶); data reduction: *SAINT*; program(s) used to solve structure: *SHELXS97* (Sheldrick, 2008 ▶); program(s) used to refine structure: *SHELXL97* (Sheldrick, 2008 ▶); molecular graphics: *ORTEP-3* (Farrugia, 1997 ▶); software used to prepare material for publication: *SHELXL97* and *PLATON* (Spek, 2009 ▶).

## Supplementary Material

Crystal structure: contains datablocks global, I. DOI: 10.1107/S1600536811000444/bt5412sup1.cif

Structure factors: contains datablocks I. DOI: 10.1107/S1600536811000444/bt5412Isup2.hkl

Additional supplementary materials:  crystallographic information; 3D view; checkCIF report

## Figures and Tables

**Table 1:** Hydrogen-bond geometry (Å, °)

*D*—H⋯*A*	*D*—H	H⋯*A*	*D*⋯*A*	*D*—H⋯*A*
C4*B*—H4*B*⋯O2*B*	0.98	2.59	3.167 (3)	118
C2*A*—H2*A*⋯O4*B*^i^	0.98	2.34	3.273 (3)	158
C19*A*—H19*A*⋯O6*B*^i^	0.93	2.49	3.360 (4)	155
C5*B*—H5*B*⋯O4*A*^ii^	0.98	2.34	3.269 (3)	157
C11*B*—H11*B*⋯O2*A*^ii^	0.93	2.53	3.404 (4)	156
C17*B*—H17*E*⋯O3*A*^iii^	0.96	2.52	3.402 (4)	153
C14*A*—H14*B*⋯O3*B*^iv^	0.96	2.52	3.402 (4)	153

Symmetry codes: (i) 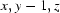; (ii) 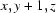; (iii) 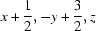; (iv) 
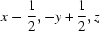
.

**Fig. 1.**
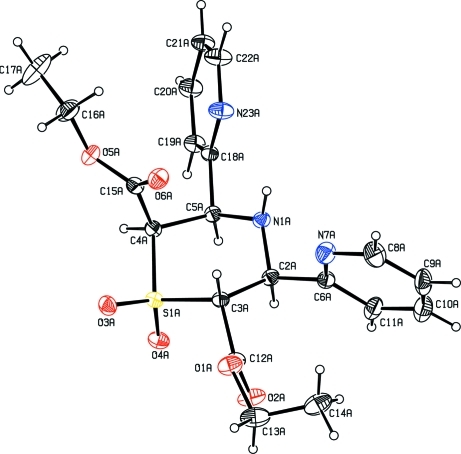
The molecular structure of the title compound, showing just one of the two molecules in the asymmetric unit. The displacement ellipsoids are drawn at the 30% probability level.

## supplementary crystallographic information

### Comment

Tetrahydro-1,4-thiazine-1,1-dioxide derivatives possess antibacterial, antifungal and antihistaminic properties (Katritzky, 1985; Ramana Reddy *et al.*, 1990; Bhaskar *et al.* 2000). Against this background and to ascertain the structure of title compound, the crystallographic studies has been carried out.

The *ORTEP* plot of the molecule is shown in Fig.1. There are two crystallographically independent molecules in the asymmetric unit. Both thiomorpholine ring systems adopt a chair conformation with the puckering parameters (Cremer & Pople, 1975) and the asymmetry parameters (Nardelli, 1983) are: for molecule A, q_2_ = 0.029 (3) Å, q_3_ = 0.598 (3) Å, φ_2_ = 41 (5)° and Δ_s_(S1A & N1A)= 2.6 (2) °; for molecule B: q_2_ = 0.039 (3) Å, q_3_ = 0.599 (3) Å, φ_2_ = 305 (4)° and Δ_s_(S1B & N1B)= 3.5 (2)°. The sum of the bond angles around the atoms N1A (346.5°) and N1B (332.2°) of the thiomorphline ring in both the molecules are in accordance with *sp^3^* hybridization.

The packing of the molecules in the crystal is stabilized by  C—H···O interactions which form a three demensional network.

### Experimental

A mixture of diethyl 2,2'-sulfonyldiacetate (1.0 mol), pyridine-2-aldehyde (2.0 mol), ammonium acetate (2.0 mol) and a few drops of alcohol were made as a homogeneous paste. Then it was stirred with 10 ml of water at room temperature for about 3 h and left overnight. The reaction mixture was diluted with excess of water. The solid that separated was filtered, washed with water and dried; crystallization from aqueous alcohol gave pure 2,6-dicarbethoxy-3,5-bis(pyridin-2-yl)tetrahydro-1,4-thiazine-1,1-dioxide (Baliah and Rangarajan, 1954).

### Refinement

H atoms were positioned geometrically (C—H=0.93–0.98 Å) and allowed to ride on their parent atoms,with *U*_iso_(H) = 1.5*U*_eq_(C) for methyl H 1.2*U*_eq_(C) for other H atoms. The amino H atoms were freely refined.

### Crystal data

**Table:**

C_20_H_23_N_3_O_6_S	*F*(000) = 1824
*M**_r_* = 433.47	*D*_x_ = 1.352 Mg m^−^^3^
Orthorhombic, *P**n**a*2_1_	Mo *K*α radiation, λ = 0.71073 Å
Hall symbol: P 2c -2n	Cell parameters from 4535 reflections
*a* = 20.7258 (13) Å	θ = 1.7–28.3°
*b* = 8.3921 (5) Å	µ = 0.19 mm^−^^1^
*c* = 24.4923 (15) Å	*T* = 293 K
*V* = 4260.0 (5) Å^3^	Block, colourless
*Z* = 8	0.20 × 0.18 × 0.16 mm

### Data collection

**Table:**

Bruker SMART APEXII area-detector diffractometer	9897 independent reflections
Radiation source: fine-focus sealed tube	7453 reflections with *I* > 2σ(*I*)
graphite	*R*_int_ = 0.027
ω and φ scans	θ_max_ = 28.3°, θ_min_ = 1.7°
Absorption correction: multi-scan (*SADABS*; Bruker, 2008)	*h* = −21→27
*T*_min_ = 0.962, *T*_max_ = 0.970	*k* = −10→11
22898 measured reflections	*l* = −32→32

### Refinement

**Table:**

Refinement on *F*^2^	Secondary atom site location: difference Fourier map
Least-squares matrix: full	Hydrogen site location: inferred from neighbouring sites
*R*[*F*^2^ > 2σ(*F*^2^)] = 0.042	H atoms treated by a mixture of independent and constrained refinement
*wR*(*F*^2^) = 0.113	*w* = 1/[σ^2^(*F*_o_^2^) + (0.0624*P*)^2^ + 0.0627*P*] where *P* = (*F*_o_^2^ + 2*F*_c_^2^)/3
*S* = 1.03	(Δ/σ)_max_ < 0.001
9897 reflections	Δρ_max_ = 0.29 e Å^−^^3^
553 parameters	Δρ_min_ = −0.17 e Å^−^^3^
1 restraint	Absolute structure: Flack (1983), 4584 Friedel pairs
Primary atom site location: structure-invariant direct methods	Flack parameter: 0.14 (6)

### Special details

**Table:**

Geometry. All e.s.d.'s (except the e.s.d. in the dihedral angle between two l.s. planes) are estimated using the full covariance matrix. The cell e.s.d.'s are taken into account individually in the estimation of e.s.d.'s in distances, angles and torsion angles; correlations between e.s.d.'s in cell parameters are only used when they are defined by crystal symmetry. An approximate (isotropic) treatment of cell e.s.d.'s is used for estimating e.s.d.'s involving l.s. planes.
Refinement. Refinement of *F*^2^ against ALL reflections. The weighted *R*-factor *wR* and goodness of fit *S* are based on *F*^2^, conventional *R*-factors *R* are based on *F*, with *F* set to zero for negative *F*^2^. The threshold expression of *F*^2^ > σ(*F*^2^) is used only for calculating *R*-factors(gt) *etc*. and is not relevant to the choice of reflections for refinement. *R*-factors based on *F*^2^ are statistically about twice as large as those based on *F*, and *R*- factors based on ALL data will be even larger.

### Fractional atomic coordinates and isotropic or equivalent isotropic displacement parameters (Å^2^)

**Table:**

	*x*	*y*	*z*	*U*_iso_*/*U*_eq_	
S1A	0.07796 (3)	0.29281 (10)	0.17383 (2)	0.03724 (14)	
O1A	−0.08829 (9)	0.1886 (3)	0.18546 (9)	0.0567 (5)	
O2A	−0.02641 (10)	0.0197 (3)	0.13848 (9)	0.0649 (6)	
O3A	0.05012 (9)	0.4478 (2)	0.16522 (8)	0.0481 (5)	
O4A	0.10104 (11)	0.2054 (3)	0.12711 (8)	0.0491 (5)	
O5A	0.17481 (10)	0.5395 (2)	0.26684 (9)	0.0538 (5)	
O6A	0.08102 (10)	0.4278 (3)	0.29336 (10)	0.0538 (6)	
N1A	0.10848 (11)	0.0586 (3)	0.27062 (12)	0.0374 (6)	
H1A	0.0979 (17)	0.092 (4)	0.3042 (15)	0.060 (11)*	
C2A	0.05581 (12)	0.0218 (3)	0.23411 (11)	0.0368 (5)	
H2A	0.0731	−0.0398	0.2034	0.044*	
C3A	0.02173 (12)	0.1721 (3)	0.21064 (10)	0.0347 (5)	
H3A	0.0037	0.2344	0.2409	0.042*	
C4A	0.14360 (13)	0.3100 (3)	0.22123 (10)	0.0364 (6)	
H4A	0.1811	0.3493	0.2010	0.044*	
C5A	0.16188 (12)	0.1416 (3)	0.24529 (11)	0.0351 (6)	
H5A	0.1774	0.0755	0.2150	0.042*	
C6A	0.00661 (13)	−0.0803 (3)	0.26320 (11)	0.0420 (6)	
C10A	−0.04476 (19)	−0.3286 (4)	0.27768 (18)	0.0736 (10)	
H10A	−0.0497	−0.4367	0.2703	0.088*	
C9A	−0.0819 (2)	−0.2545 (4)	0.3169 (3)	0.0729 (17)	
H9A	−0.1135	−0.3112	0.3356	0.088*	
C8A	−0.07184 (18)	−0.0963 (5)	0.32788 (15)	0.0717 (10)	
H8A	−0.0965	−0.0486	0.3551	0.086*	
N7A	−0.02839 (13)	−0.0069 (3)	0.30152 (10)	0.0577 (7)	
C12A	−0.03341 (13)	0.1183 (3)	0.17295 (12)	0.0440 (6)	
C13A	−0.14618 (16)	0.1198 (5)	0.16069 (16)	0.0735 (11)	
H13A	−0.1359	0.0752	0.1252	0.088*	
H13B	−0.1789	0.2013	0.1559	0.088*	
C14A	−0.17102 (18)	−0.0106 (5)	0.19873 (18)	0.0800 (11)	
H14A	−0.1410	−0.0978	0.1991	0.120*	
H14B	−0.2122	−0.0475	0.1860	0.120*	
H14C	−0.1755	0.0314	0.2350	0.120*	
C15A	0.12790 (15)	0.4312 (4)	0.26447 (14)	0.0424 (7)	
C16A	0.16830 (19)	0.6658 (4)	0.30708 (18)	0.0735 (11)	
H16A	0.1500	0.6239	0.3406	0.088*	
H16B	0.1401	0.7489	0.2934	0.088*	
C17A	0.2328 (3)	0.7300 (6)	0.3173 (3)	0.115 (2)	
H17A	0.2590	0.6500	0.3344	0.172*	
H17B	0.2297	0.8211	0.3408	0.172*	
H17C	0.2522	0.7611	0.2833	0.172*	
C18A	0.21738 (12)	0.1620 (3)	0.28544 (10)	0.0354 (5)	
C19A	0.28099 (13)	0.1448 (3)	0.26942 (12)	0.0452 (6)	
H19A	0.2910	0.1166	0.2337	0.054*	
C20A	0.32921 (14)	0.1699 (4)	0.30700 (13)	0.0557 (8)	
H20A	0.3722	0.1605	0.2967	0.067*	
C21A	0.31343 (18)	0.2091 (5)	0.36023 (16)	0.0567 (8)	
H21A	0.3450	0.2258	0.3866	0.068*	
C22A	0.24882 (18)	0.2220 (6)	0.3722 (2)	0.0659 (12)	
H22A	0.2376	0.2470	0.4080	0.079*	
C11A	−0.00059 (17)	−0.2393 (4)	0.2500 (2)	0.0585 (13)	
H11A	0.0244	−0.2849	0.2226	0.070*	
N23A	0.20096 (12)	0.2015 (4)	0.33639 (12)	0.0539 (6)	
S1B	0.16804 (3)	0.70742 (10)	0.10968 (2)	0.03664 (14)	
O1B	0.07041 (10)	0.4650 (2)	0.01390 (9)	0.0517 (5)	
O2B	0.16626 (10)	0.5726 (3)	−0.00902 (9)	0.0503 (6)	
O3B	0.19608 (9)	0.5531 (2)	0.11852 (8)	0.0470 (5)	
O4B	0.14445 (11)	0.7948 (3)	0.15549 (8)	0.0495 (5)	
O5B	0.33391 (9)	0.8084 (3)	0.09752 (8)	0.0561 (5)	
O6B	0.27294 (11)	0.9824 (3)	0.14422 (9)	0.0645 (6)	
N1B	0.13666 (12)	0.9403 (3)	0.01219 (12)	0.0375 (6)	
H1B	0.1533 (11)	0.885 (3)	−0.0151 (10)	0.016 (6)*	
C2B	0.08363 (12)	0.8566 (3)	0.03772 (11)	0.0364 (6)	
H2B	0.0675	0.9231	0.0676	0.044*	
C3B	0.10241 (12)	0.6909 (3)	0.06219 (11)	0.0350 (5)	
H3B	0.0649	0.6509	0.0824	0.042*	
C4B	0.22467 (12)	0.8277 (3)	0.07248 (10)	0.0360 (5)	
H4B	0.2426	0.7655	0.0422	0.043*	
C5B	0.18996 (12)	0.9771 (3)	0.04946 (10)	0.0368 (5)	
H5B	0.1725	1.0379	0.0803	0.044*	
C6B	0.02908 (13)	0.8343 (3)	−0.00298 (10)	0.0379 (6)	
N7B	0.04684 (13)	0.7879 (4)	−0.05309 (11)	0.0515 (6)	
C8B	−0.00031 (19)	0.7602 (4)	−0.0897 (2)	0.0568 (11)	
H8B	0.0112	0.7298	−0.1249	0.068*	
C9B	−0.0645 (2)	0.7749 (5)	−0.07749 (18)	0.0585 (10)	
H9B	−0.0957	0.7527	−0.1037	0.070*	
C10B	−0.08203 (15)	0.8230 (4)	−0.02585 (14)	0.0591 (8)	
H10B	−0.1253	0.8341	−0.0166	0.071*	
C11B	−0.03454 (13)	0.8544 (4)	0.01173 (12)	0.0488 (7)	
H11B	−0.0452	0.8888	0.0467	0.059*	
C12B	0.11816 (14)	0.5696 (4)	0.01801 (13)	0.0367 (6)	
C13B	0.07553 (18)	0.3485 (4)	−0.02997 (18)	0.0738 (11)	
H13C	0.1073	0.2679	−0.0208	0.089*	
H13D	0.0886	0.4002	−0.0636	0.089*	
C14B	0.0110 (2)	0.2750 (6)	−0.0364 (2)	0.1052 (19)	
H14D	−0.0049	0.2424	−0.0014	0.158*	
H14E	0.0142	0.1838	−0.0600	0.158*	
H14F	−0.0181	0.3512	−0.0522	0.158*	
C15B	0.27901 (12)	0.8812 (3)	0.10983 (11)	0.0425 (6)	
C16B	0.39240 (16)	0.8787 (5)	0.12212 (16)	0.0777 (12)	
H16C	0.4253	0.7975	0.1264	0.093*	
H16D	0.3824	0.9215	0.1579	0.093*	
C17B	0.41688 (19)	1.0101 (5)	0.08536 (18)	0.0843 (12)	
H17D	0.4262	0.9672	0.0499	0.126*	
H17E	0.4554	1.0550	0.1007	0.126*	
H17F	0.3846	1.0915	0.0822	0.126*	
C18B	0.23894 (13)	1.0806 (3)	0.02030 (11)	0.0411 (6)	
N23B	0.27697 (14)	1.0074 (3)	−0.01583 (10)	0.0572 (7)	
C22B	0.32086 (19)	1.0969 (4)	−0.04148 (16)	0.0744 (10)	
H22B	0.3487	1.0465	−0.0658	0.089*	
C21B	0.3276 (2)	1.2571 (5)	−0.0344 (2)	0.0787 (17)	
H21B	0.3576	1.3150	−0.0545	0.094*	
C20B	0.28872 (18)	1.3298 (4)	0.0032 (2)	0.0831 (13)	
H20B	0.2926	1.4386	0.0097	0.100*	
C19B	0.24368 (18)	1.2407 (4)	0.0315 (2)	0.0593 (13)	
H19B	0.2172	1.2880	0.0575	0.071*	

### Atomic displacement parameters (Å^2^)

**Table:**

	*U*^11^	*U*^22^	*U*^33^	*U*^12^	*U*^13^	*U*^23^
S1A	0.0374 (3)	0.0421 (3)	0.0323 (3)	0.0025 (3)	−0.0030 (3)	0.0002 (4)
O1A	0.0377 (11)	0.0668 (13)	0.0655 (14)	0.0079 (10)	−0.0082 (10)	−0.0139 (11)
O2A	0.0520 (13)	0.0804 (15)	0.0622 (13)	0.0050 (12)	−0.0126 (11)	−0.0321 (12)
O3A	0.0476 (11)	0.0430 (10)	0.0537 (12)	0.0053 (8)	−0.0067 (9)	0.0093 (9)
O4A	0.0532 (12)	0.0603 (11)	0.0337 (11)	0.0042 (12)	0.0014 (9)	−0.0040 (11)
O5A	0.0589 (13)	0.0410 (11)	0.0614 (12)	−0.0130 (9)	−0.0042 (10)	−0.0063 (10)
O6A	0.0538 (13)	0.0502 (13)	0.0574 (13)	0.0001 (10)	0.0068 (11)	−0.0100 (11)
N1A	0.0323 (12)	0.0400 (13)	0.0399 (14)	−0.0009 (11)	−0.0043 (11)	−0.0006 (11)
C2A	0.0352 (13)	0.0345 (13)	0.0408 (13)	0.0022 (11)	−0.0021 (11)	−0.0047 (11)
C3A	0.0324 (13)	0.0374 (13)	0.0344 (12)	0.0013 (10)	−0.0024 (10)	−0.0018 (10)
C4A	0.0323 (14)	0.0416 (16)	0.0352 (14)	−0.0007 (12)	−0.0001 (10)	0.0036 (12)
C5A	0.0349 (14)	0.0346 (14)	0.0358 (13)	0.0015 (11)	0.0006 (11)	−0.0025 (11)
C6A	0.0368 (14)	0.0416 (14)	0.0475 (15)	−0.0005 (12)	−0.0081 (11)	0.0030 (12)
C10A	0.065 (2)	0.0521 (19)	0.103 (3)	−0.0119 (18)	−0.005 (2)	0.013 (2)
C9A	0.050 (2)	0.063 (3)	0.106 (4)	−0.0172 (17)	−0.009 (2)	0.035 (2)
C8A	0.067 (2)	0.080 (2)	0.068 (2)	0.002 (2)	0.0171 (18)	0.0119 (19)
N7A	0.0614 (17)	0.0557 (15)	0.0561 (15)	−0.0071 (13)	0.0131 (13)	−0.0039 (12)
C12A	0.0407 (15)	0.0507 (15)	0.0405 (13)	0.0041 (12)	−0.0055 (12)	−0.0069 (13)
C13A	0.0383 (17)	0.107 (3)	0.075 (2)	0.0097 (19)	−0.0178 (16)	−0.019 (2)
C14A	0.050 (2)	0.093 (3)	0.097 (3)	−0.012 (2)	−0.016 (2)	−0.020 (2)
C15A	0.0462 (17)	0.0414 (16)	0.0396 (16)	0.0018 (14)	−0.0072 (14)	0.0037 (13)
C16A	0.082 (3)	0.0501 (19)	0.089 (3)	−0.0045 (17)	−0.019 (2)	−0.0198 (18)
C17A	0.092 (3)	0.075 (3)	0.177 (7)	−0.033 (3)	0.002 (4)	−0.051 (3)
C18A	0.0331 (13)	0.0388 (12)	0.0343 (12)	0.0019 (11)	−0.0024 (10)	−0.0006 (10)
C19A	0.0363 (14)	0.0587 (16)	0.0405 (14)	0.0039 (13)	0.0010 (11)	−0.0047 (13)
C20A	0.0342 (15)	0.077 (2)	0.0561 (18)	0.0046 (15)	−0.0052 (13)	−0.0026 (15)
C21A	0.0467 (19)	0.073 (2)	0.050 (2)	0.0023 (18)	−0.0194 (16)	−0.0008 (19)
C22A	0.055 (3)	0.102 (3)	0.040 (2)	0.0021 (19)	−0.0054 (17)	−0.0149 (19)
C11A	0.054 (2)	0.039 (2)	0.082 (4)	−0.0049 (13)	−0.007 (2)	−0.0010 (15)
N23A	0.0359 (14)	0.0854 (18)	0.0403 (14)	0.0052 (15)	−0.0017 (11)	−0.0102 (16)
S1B	0.0379 (4)	0.0414 (3)	0.0307 (3)	0.0043 (3)	−0.0034 (3)	0.0005 (4)
O1B	0.0532 (12)	0.0473 (12)	0.0547 (12)	−0.0092 (9)	0.0001 (10)	−0.0088 (10)
O2B	0.0460 (13)	0.0588 (13)	0.0463 (12)	0.0013 (10)	0.0042 (10)	−0.0114 (11)
O3B	0.0485 (11)	0.0453 (10)	0.0472 (11)	0.0073 (8)	−0.0081 (9)	0.0048 (9)
O4B	0.0553 (13)	0.0604 (11)	0.0329 (10)	0.0066 (12)	0.0027 (9)	−0.0046 (11)
O5B	0.0375 (11)	0.0719 (14)	0.0589 (14)	0.0093 (11)	−0.0105 (9)	−0.0063 (11)
O6B	0.0548 (13)	0.0805 (15)	0.0582 (12)	0.0028 (12)	−0.0105 (10)	−0.0314 (12)
N1B	0.0380 (13)	0.0373 (13)	0.0374 (14)	0.0029 (11)	−0.0031 (11)	0.0001 (11)
C2B	0.0320 (13)	0.0415 (15)	0.0358 (14)	0.0041 (11)	−0.0027 (11)	−0.0024 (11)
C3B	0.0305 (13)	0.0410 (16)	0.0335 (13)	−0.0004 (12)	0.0005 (10)	0.0004 (11)
C4B	0.0348 (14)	0.0408 (14)	0.0325 (12)	0.0037 (11)	−0.0002 (10)	−0.0062 (10)
C5B	0.0364 (14)	0.0391 (13)	0.0351 (13)	0.0004 (11)	−0.0025 (11)	−0.0045 (11)
C6B	0.0371 (14)	0.0376 (13)	0.0390 (13)	0.0024 (11)	−0.0027 (11)	0.0065 (10)
N7B	0.0429 (15)	0.0785 (16)	0.0332 (13)	0.0011 (14)	−0.0019 (10)	0.0008 (14)
C8B	0.057 (2)	0.082 (3)	0.032 (2)	0.0012 (15)	−0.0099 (16)	0.0009 (13)
C9B	0.049 (2)	0.077 (2)	0.050 (2)	−0.0088 (18)	−0.0166 (16)	0.0103 (18)
C10B	0.0347 (16)	0.076 (2)	0.066 (2)	0.0028 (15)	−0.0032 (15)	0.0044 (16)
C11B	0.0404 (15)	0.0606 (18)	0.0454 (15)	0.0073 (13)	−0.0001 (13)	−0.0013 (13)
C12B	0.0385 (15)	0.0351 (14)	0.0366 (14)	0.0026 (13)	−0.0081 (13)	0.0010 (11)
C13B	0.076 (3)	0.060 (2)	0.086 (3)	−0.0058 (18)	−0.011 (2)	−0.0311 (19)
C14B	0.095 (4)	0.110 (4)	0.111 (5)	−0.034 (3)	−0.005 (3)	−0.047 (3)
C15B	0.0384 (14)	0.0501 (15)	0.0390 (13)	0.0035 (11)	−0.0026 (12)	−0.0004 (13)
C16B	0.0412 (18)	0.117 (3)	0.075 (2)	0.013 (2)	−0.0250 (17)	−0.020 (2)
C17B	0.053 (2)	0.102 (3)	0.099 (3)	−0.018 (2)	−0.011 (2)	−0.019 (3)
C18B	0.0384 (14)	0.0385 (13)	0.0464 (15)	−0.0006 (11)	−0.0063 (12)	−0.0011 (12)
N23B	0.0644 (18)	0.0517 (14)	0.0557 (15)	−0.0036 (13)	0.0154 (13)	0.0002 (12)
C22B	0.073 (2)	0.072 (2)	0.079 (2)	−0.002 (2)	0.026 (2)	0.015 (2)
C21B	0.063 (3)	0.081 (4)	0.093 (4)	−0.010 (2)	0.014 (3)	0.033 (2)
C20B	0.059 (2)	0.0443 (18)	0.146 (4)	−0.0100 (17)	0.003 (3)	0.014 (2)
C19B	0.046 (2)	0.045 (2)	0.087 (4)	0.0008 (13)	0.002 (2)	−0.0031 (15)

### Geometric parameters (Å, °)

**Table:**

S1A—O3A	1.439 (2)	S1B—O4B	1.427 (2)
S1A—O4A	1.441 (2)	S1B—O3B	1.4356 (19)
S1A—C3A	1.788 (3)	S1B—C3B	1.795 (3)
S1A—C4A	1.794 (3)	S1B—C4B	1.796 (3)
O1A—C12A	1.318 (3)	O1B—C12B	1.327 (3)
O1A—C13A	1.463 (4)	O1B—C13B	1.456 (4)
O2A—C12A	1.191 (3)	O2B—C12B	1.197 (3)
O5A—C15A	1.332 (4)	O5B—C15B	1.326 (3)
O5A—C16A	1.454 (4)	O5B—C16B	1.477 (4)
O6A—C15A	1.202 (4)	O6B—C15B	1.202 (3)
N1A—C2A	1.445 (4)	N1B—C2B	1.447 (4)
N1A—C5A	1.447 (3)	N1B—C5B	1.466 (4)
N1A—H1A	0.90 (4)	N1B—H1B	0.88 (2)
C2A—C6A	1.511 (4)	C2B—C6B	1.519 (4)
C2A—C3A	1.556 (4)	C2B—C3B	1.563 (4)
C2A—H2A	0.9800	C2B—H2B	0.9800
C3A—C12A	1.537 (4)	C3B—C12B	1.521 (4)
C3A—H3A	0.9800	C3B—H3B	0.9800
C4A—C15A	1.504 (4)	C4B—C15B	1.519 (4)
C4A—C5A	1.577 (4)	C4B—C5B	1.552 (4)
C4A—H4A	0.9800	C4B—H4B	0.9800
C5A—C18A	1.523 (3)	C5B—C18B	1.515 (4)
C5A—H5A	0.9800	C5B—H5B	0.9800
C6A—N7A	1.337 (4)	C6B—N7B	1.339 (4)
C6A—C11A	1.381 (4)	C6B—C11B	1.377 (4)
C10A—C11A	1.364 (5)	N7B—C8B	1.346 (5)
C10A—C9A	1.379 (6)	C8B—C9B	1.368 (5)
C10A—H10A	0.9300	C8B—H8B	0.9300
C9A—C8A	1.371 (5)	C9B—C10B	1.377 (6)
C9A—H9A	0.9300	C9B—H9B	0.9300
C8A—N7A	1.338 (4)	C10B—C11B	1.373 (4)
C8A—H8A	0.9300	C10B—H10B	0.9300
C13A—C14A	1.527 (6)	C11B—H11B	0.9300
C13A—H13A	0.9700	C13B—C14B	1.481 (5)
C13A—H13B	0.9700	C13B—H13C	0.9700
C14A—H14A	0.9600	C13B—H13D	0.9700
C14A—H14B	0.9600	C14B—H14D	0.9600
C14A—H14C	0.9600	C14B—H14E	0.9600
C16A—C17A	1.464 (6)	C14B—H14F	0.9600
C16A—H16A	0.9700	C16B—C17B	1.511 (6)
C16A—H16B	0.9700	C16B—H16C	0.9700
C17A—H17A	0.9600	C16B—H16D	0.9700
C17A—H17B	0.9600	C17B—H17D	0.9600
C17A—H17C	0.9600	C17B—H17E	0.9600
C18A—N23A	1.335 (4)	C17B—H17F	0.9600
C18A—C19A	1.383 (4)	C18B—N23B	1.335 (4)
C19A—C20A	1.375 (4)	C18B—C19B	1.375 (4)
C19A—H19A	0.9300	N23B—C22B	1.336 (4)
C20A—C21A	1.384 (5)	C22B—C21B	1.362 (5)
C20A—H20A	0.9300	C22B—H22B	0.9300
C21A—C22A	1.375 (5)	C21B—C20B	1.367 (6)
C21A—H21A	0.9300	C21B—H21B	0.9300
C22A—N23A	1.335 (5)	C20B—C19B	1.382 (5)
C22A—H22A	0.9300	C20B—H20B	0.9300
C11A—H11A	0.9300	C19B—H19B	0.9300
			
O3A—S1A—O4A	118.48 (13)	O4B—S1B—O3B	118.95 (13)
O3A—S1A—C3A	108.96 (12)	O4B—S1B—C3B	106.83 (13)
O4A—S1A—C3A	109.18 (13)	O3B—S1B—C3B	109.55 (13)
O3A—S1A—C4A	109.05 (13)	O4B—S1B—C4B	109.49 (13)
O4A—S1A—C4A	107.63 (13)	O3B—S1B—C4B	108.60 (12)
C3A—S1A—C4A	102.32 (12)	C3B—S1B—C4B	102.12 (12)
C12A—O1A—C13A	115.8 (2)	C12B—O1B—C13B	116.5 (2)
C15A—O5A—C16A	117.4 (3)	C15B—O5B—C16B	115.3 (2)
C2A—N1A—C5A	114.5 (2)	C2B—N1B—C5B	113.9 (2)
C2A—N1A—H1A	117 (2)	C2B—N1B—H1B	111.6 (15)
C5A—N1A—H1A	115 (2)	C5B—N1B—H1B	106.7 (15)
N1A—C2A—C6A	109.8 (2)	N1B—C2B—C6B	110.0 (2)
N1A—C2A—C3A	113.5 (2)	N1B—C2B—C3B	114.1 (2)
C6A—C2A—C3A	109.1 (2)	C6B—C2B—C3B	109.1 (2)
N1A—C2A—H2A	108.1	N1B—C2B—H2B	107.8
C6A—C2A—H2A	108.1	C6B—C2B—H2B	107.8
C3A—C2A—H2A	108.1	C3B—C2B—H2B	107.8
C12A—C3A—C2A	108.8 (2)	C12B—C3B—C2B	112.1 (2)
C12A—C3A—S1A	110.37 (18)	C12B—C3B—S1B	110.50 (18)
C2A—C3A—S1A	110.48 (17)	C2B—C3B—S1B	111.6 (2)
C12A—C3A—H3A	109.1	C12B—C3B—H3B	107.5
C2A—C3A—H3A	109.1	C2B—C3B—H3B	107.5
S1A—C3A—H3A	109.1	S1B—C3B—H3B	107.5
C15A—C4A—C5A	113.3 (2)	C15B—C4B—C5B	108.9 (2)
C15A—C4A—S1A	110.23 (19)	C15B—C4B—S1B	110.20 (18)
C5A—C4A—S1A	110.61 (19)	C5B—C4B—S1B	109.61 (17)
C15A—C4A—H4A	107.5	C15B—C4B—H4B	109.4
C5A—C4A—H4A	107.5	C5B—C4B—H4B	109.4
S1A—C4A—H4A	107.5	S1B—C4B—H4B	109.4
N1A—C5A—C18A	110.8 (2)	N1B—C5B—C18B	109.4 (2)
N1A—C5A—C4A	114.1 (2)	N1B—C5B—C4B	113.9 (2)
C18A—C5A—C4A	108.8 (2)	C18B—C5B—C4B	108.9 (2)
N1A—C5A—H5A	107.7	N1B—C5B—H5B	108.1
C18A—C5A—H5A	107.7	C18B—C5B—H5B	108.1
C4A—C5A—H5A	107.7	C4B—C5B—H5B	108.1
N7A—C6A—C11A	123.4 (3)	N7B—C6B—C11B	122.6 (3)
N7A—C6A—C2A	115.9 (2)	N7B—C6B—C2B	115.6 (2)
C11A—C6A—C2A	120.7 (3)	C11B—C6B—C2B	121.7 (2)
C11A—C10A—C9A	118.2 (4)	C6B—N7B—C8B	117.4 (3)
C11A—C10A—H10A	120.9	N7B—C8B—C9B	123.0 (4)
C9A—C10A—H10A	120.9	N7B—C8B—H8B	118.5
C8A—C9A—C10A	119.2 (4)	C9B—C8B—H8B	118.5
C8A—C9A—H9A	120.4	C8B—C9B—C10B	118.9 (4)
C10A—C9A—H9A	120.4	C8B—C9B—H9B	120.5
N7A—C8A—C9A	123.4 (4)	C10B—C9B—H9B	120.5
N7A—C8A—H8A	118.3	C11B—C10B—C9B	118.9 (3)
C9A—C8A—H8A	118.3	C11B—C10B—H10B	120.6
C6A—N7A—C8A	116.5 (3)	C9B—C10B—H10B	120.6
O2A—C12A—O1A	125.5 (3)	C10B—C11B—C6B	119.2 (3)
O2A—C12A—C3A	122.6 (2)	C10B—C11B—H11B	120.4
O1A—C12A—C3A	111.8 (2)	C6B—C11B—H11B	120.4
O1A—C13A—C14A	107.9 (3)	O2B—C12B—O1B	126.4 (3)
O1A—C13A—H13A	110.1	O2B—C12B—C3B	123.9 (3)
C14A—C13A—H13A	110.1	O1B—C12B—C3B	109.7 (2)
O1A—C13A—H13B	110.1	O1B—C13B—C14B	107.0 (3)
C14A—C13A—H13B	110.1	O1B—C13B—H13C	110.3
H13A—C13A—H13B	108.4	C14B—C13B—H13C	110.3
C13A—C14A—H14A	109.5	O1B—C13B—H13D	110.3
C13A—C14A—H14B	109.5	C14B—C13B—H13D	110.3
H14A—C14A—H14B	109.5	H13C—C13B—H13D	108.6
C13A—C14A—H14C	109.5	C13B—C14B—H14D	109.5
H14A—C14A—H14C	109.5	C13B—C14B—H14E	109.5
H14B—C14A—H14C	109.5	H14D—C14B—H14E	109.5
O6A—C15A—O5A	125.4 (3)	C13B—C14B—H14F	109.5
O6A—C15A—C4A	125.0 (3)	H14D—C14B—H14F	109.5
O5A—C15A—C4A	109.5 (3)	H14E—C14B—H14F	109.5
O5A—C16A—C17A	107.4 (4)	O6B—C15B—O5B	125.0 (3)
O5A—C16A—H16A	110.2	O6B—C15B—C4B	123.6 (2)
C17A—C16A—H16A	110.2	O5B—C15B—C4B	111.3 (2)
O5A—C16A—H16B	110.2	O5B—C16B—C17B	108.9 (3)
C17A—C16A—H16B	110.2	O5B—C16B—H16C	109.9
H16A—C16A—H16B	108.5	C17B—C16B—H16C	109.9
C16A—C17A—H17A	109.5	O5B—C16B—H16D	109.9
C16A—C17A—H17B	109.5	C17B—C16B—H16D	109.9
H17A—C17A—H17B	109.5	H16C—C16B—H16D	108.3
C16A—C17A—H17C	109.5	C16B—C17B—H17D	109.5
H17A—C17A—H17C	109.5	C16B—C17B—H17E	109.5
H17B—C17A—H17C	109.5	H17D—C17B—H17E	109.5
N23A—C18A—C19A	122.3 (2)	C16B—C17B—H17F	109.5
N23A—C18A—C5A	116.0 (2)	H17D—C17B—H17F	109.5
C19A—C18A—C5A	121.7 (2)	H17E—C17B—H17F	109.5
C20A—C19A—C18A	119.1 (3)	N23B—C18B—C19B	122.6 (3)
C20A—C19A—H19A	120.4	N23B—C18B—C5B	116.4 (2)
C18A—C19A—H19A	120.4	C19B—C18B—C5B	121.0 (3)
C19A—C20A—C21A	119.7 (3)	C18B—N23B—C22B	117.1 (3)
C19A—C20A—H20A	120.2	N23B—C22B—C21B	124.4 (4)
C21A—C20A—H20A	120.2	N23B—C22B—H22B	117.8
C22A—C21A—C20A	116.7 (4)	C21B—C22B—H22B	117.8
C22A—C21A—H21A	121.6	C22B—C21B—C20B	117.8 (4)
C20A—C21A—H21A	121.6	C22B—C21B—H21B	121.1
N23A—C22A—C21A	125.0 (4)	C20B—C21B—H21B	121.1
N23A—C22A—H22A	117.5	C21B—C20B—C19B	119.6 (3)
C21A—C22A—H22A	117.5	C21B—C20B—H20B	120.2
C10A—C11A—C6A	119.1 (4)	C19B—C20B—H20B	120.2
C10A—C11A—H11A	120.4	C18B—C19B—C20B	118.5 (4)
C6A—C11A—H11A	120.4	C18B—C19B—H19B	120.7
C18A—N23A—C22A	117.2 (3)	C20B—C19B—H19B	120.7
			
C5A—N1A—C2A—C6A	173.6 (2)	C5B—N1B—C2B—C6B	176.0 (2)
C5A—N1A—C2A—C3A	−63.9 (3)	C5B—N1B—C2B—C3B	−61.0 (3)
N1A—C2A—C3A—C12A	−179.6 (2)	N1B—C2B—C3B—C12B	−69.3 (3)
C6A—C2A—C3A—C12A	−56.7 (3)	C6B—C2B—C3B—C12B	54.2 (3)
N1A—C2A—C3A—S1A	59.1 (3)	N1B—C2B—C3B—S1B	55.2 (3)
C6A—C2A—C3A—S1A	−178.03 (17)	C6B—C2B—C3B—S1B	178.71 (17)
O3A—S1A—C3A—C12A	74.7 (2)	O4B—S1B—C3B—C12B	−167.6 (2)
O4A—S1A—C3A—C12A	−56.1 (2)	O3B—S1B—C3B—C12B	−37.5 (2)
C4A—S1A—C3A—C12A	−169.94 (18)	C4B—S1B—C3B—C12B	77.5 (2)
O3A—S1A—C3A—C2A	−164.99 (17)	O4B—S1B—C3B—C2B	67.0 (2)
O4A—S1A—C3A—C2A	64.2 (2)	O3B—S1B—C3B—C2B	−162.92 (17)
C4A—S1A—C3A—C2A	−49.63 (19)	C4B—S1B—C3B—C2B	−47.94 (19)
O3A—S1A—C4A—C15A	37.0 (2)	O4B—S1B—C4B—C15B	56.6 (2)
O4A—S1A—C4A—C15A	166.8 (2)	O3B—S1B—C4B—C15B	−74.7 (2)
C3A—S1A—C4A—C15A	−78.3 (2)	C3B—S1B—C4B—C15B	169.59 (18)
O3A—S1A—C4A—C5A	163.07 (17)	O4B—S1B—C4B—C5B	−63.2 (2)
O4A—S1A—C4A—C5A	−67.2 (2)	O3B—S1B—C4B—C5B	165.46 (16)
C3A—S1A—C4A—C5A	47.8 (2)	C3B—S1B—C4B—C5B	49.78 (19)
C2A—N1A—C5A—C18A	−174.9 (2)	C2B—N1B—C5B—C18B	−173.5 (2)
C2A—N1A—C5A—C4A	62.0 (3)	C2B—N1B—C5B—C4B	64.3 (3)
C15A—C4A—C5A—N1A	68.8 (3)	C15B—C4B—C5B—N1B	179.4 (2)
S1A—C4A—C5A—N1A	−55.5 (3)	S1B—C4B—C5B—N1B	−60.0 (2)
C15A—C4A—C5A—C18A	−55.4 (3)	C15B—C4B—C5B—C18B	57.0 (3)
S1A—C4A—C5A—C18A	−179.67 (17)	S1B—C4B—C5B—C18B	177.62 (17)
N1A—C2A—C6A—N7A	71.3 (3)	N1B—C2B—C6B—N7B	43.8 (3)
C3A—C2A—C6A—N7A	−53.7 (3)	C3B—C2B—C6B—N7B	−82.1 (3)
N1A—C2A—C6A—C11A	−108.8 (3)	N1B—C2B—C6B—C11B	−138.7 (3)
C3A—C2A—C6A—C11A	126.3 (3)	C3B—C2B—C6B—C11B	95.4 (3)
C11A—C10A—C9A—C8A	−2.2 (7)	C11B—C6B—N7B—C8B	−0.2 (5)
C10A—C9A—C8A—N7A	1.7 (7)	C2B—C6B—N7B—C8B	177.2 (3)
C11A—C6A—N7A—C8A	0.5 (5)	C6B—N7B—C8B—C9B	−1.1 (5)
C2A—C6A—N7A—C8A	−179.5 (3)	N7B—C8B—C9B—C10B	1.3 (6)
C9A—C8A—N7A—C6A	−0.8 (5)	C8B—C9B—C10B—C11B	−0.1 (5)
C13A—O1A—C12A—O2A	10.4 (5)	C9B—C10B—C11B—C6B	−1.1 (5)
C13A—O1A—C12A—C3A	−166.5 (3)	N7B—C6B—C11B—C10B	1.3 (4)
C2A—C3A—C12A—O2A	−48.3 (4)	C2B—C6B—C11B—C10B	−176.0 (3)
S1A—C3A—C12A—O2A	73.0 (3)	C13B—O1B—C12B—O2B	−3.9 (5)
C2A—C3A—C12A—O1A	128.7 (2)	C13B—O1B—C12B—C3B	174.9 (3)
S1A—C3A—C12A—O1A	−109.9 (2)	C2B—C3B—C12B—O2B	72.3 (4)
C12A—O1A—C13A—C14A	88.6 (3)	S1B—C3B—C12B—O2B	−52.8 (4)
C16A—O5A—C15A—O6A	−1.1 (5)	C2B—C3B—C12B—O1B	−106.4 (3)
C16A—O5A—C15A—C4A	−179.2 (3)	S1B—C3B—C12B—O1B	128.4 (2)
C5A—C4A—C15A—O6A	−68.7 (4)	C12B—O1B—C13B—C14B	−165.0 (4)
S1A—C4A—C15A—O6A	55.8 (4)	C16B—O5B—C15B—O6B	−10.6 (4)
C5A—C4A—C15A—O5A	109.4 (3)	C16B—O5B—C15B—C4B	166.0 (3)
S1A—C4A—C15A—O5A	−126.1 (2)	C5B—C4B—C15B—O6B	46.4 (3)
C15A—O5A—C16A—C17A	158.4 (4)	S1B—C4B—C15B—O6B	−73.8 (3)
N1A—C5A—C18A—N23A	−41.2 (3)	C5B—C4B—C15B—O5B	−130.3 (2)
C4A—C5A—C18A—N23A	84.9 (3)	S1B—C4B—C15B—O5B	109.5 (2)
N1A—C5A—C18A—C19A	140.5 (3)	C15B—O5B—C16B—C17B	−86.6 (3)
C4A—C5A—C18A—C19A	−93.4 (3)	N1B—C5B—C18B—N23B	−75.4 (3)
N23A—C18A—C19A—C20A	−0.4 (4)	C4B—C5B—C18B—N23B	49.7 (3)
C5A—C18A—C19A—C20A	177.8 (3)	N1B—C5B—C18B—C19B	105.2 (4)
C18A—C19A—C20A—C21A	1.1 (5)	C4B—C5B—C18B—C19B	−129.7 (3)
C19A—C20A—C21A—C22A	−0.5 (6)	C19B—C18B—N23B—C22B	0.0 (5)
C20A—C21A—C22A—N23A	−0.8 (7)	C5B—C18B—N23B—C22B	−179.3 (3)
C9A—C10A—C11A—C6A	1.9 (6)	C18B—N23B—C22B—C21B	−2.3 (6)
N7A—C6A—C11A—C10A	−1.1 (6)	N23B—C22B—C21B—C20B	3.0 (8)
C2A—C6A—C11A—C10A	179.0 (3)	C22B—C21B—C20B—C19B	−1.3 (7)
C19A—C18A—N23A—C22A	−0.9 (5)	N23B—C18B—C19B—C20B	1.5 (6)
C5A—C18A—N23A—C22A	−179.2 (3)	C5B—C18B—C19B—C20B	−179.3 (3)
C21A—C22A—N23A—C18A	1.5 (7)	C21B—C20B—C19B—C18B	−0.7 (7)

### Hydrogen-bond geometry (Å, °)

**Table:**

*D*—H···*A*	*D*—H	H···*A*	*D*···*A*	*D*—H···*A*
C4B—H4B···O2B	0.98	2.59	3.167 (3)	118
C2A—H2A···O4B^i^	0.98	2.34	3.273 (3)	158
C19A—H19A···O6B^i^	0.93	2.49	3.360 (4)	155
C5B—H5B···O4A^ii^	0.98	2.34	3.269 (3)	157
C11B—H11B···O2A^ii^	0.93	2.53	3.404 (4)	156
C17B—H17E···O3A^iii^	0.96	2.52	3.402 (4)	153
C14A—H14B···O3B^iv^	0.96	2.52	3.402 (4)	153

Symmetry codes:  (i) *x*, *y*−1, *z*; (ii) *x*, *y*+1, *z*; (iii) *x*+1/2, −*y*+3/2, *z*; (iv) *x*−1/2, −*y*+1/2, *z*.
